# Management of a partially ankylosed mandibular canine using controlled luxation, serial intraoral scan superimposition, and a customized lingual bracket: A case report

**DOI:** 10.1097/MD.0000000000049869

**Published:** 2026-07-24

**Authors:** Viet Anh Nguyen, Thi Hanh Pham, Tim Joda, Hang Nga Mai, Viet Hoang, Anand Marya

**Affiliations:** a School of Dentistry, Hanoi Medical University, Hanoi, Vietnam; b Private Practice, Viet Anh Orthodontic Clinic, Hanoi, Vietnam; c Center for Dental Medicine, University of Zurich, Zurich, Switzerland; d Institute for Translational Research in Dentistry, Kyungpook National University, Daegu, Korea; e Digital Industrial Design and Manufacturing Research Unit, Faculty of Engineering at Sriracha, Kasetsart University, Sriracha, Chonburi, Thailand; f Department of Orthodontics, Faculty of Dentistry, University of Puthisastra, Phnom Penh, Cambodia.

**Keywords:** bracket debonding, dentistry, digital monitoring, orthodontics, surgical orthodontics, tooth movement

## Abstract

**Rationale:**

Tooth ankylosis is difficult to diagnose and monitor because clinical signs are nonspecific, and radiographs provide only static information.

**Patient concerns:**

An adult female was referred after prolonged orthodontic failure involving a nonresponsive mandibular left canine, severe anterior open bite, negative overjet, lower midline deviation, and repeated debonding of a stock lingual bracket.

**Diagnoses:**

The patient was diagnosed with partial ankylosis of the mandibular left canine, likely related to previous segmental osteotomy and fixation-screw proximity, together with a dental Class III relationship on a skeletal Class II base.

**Interventions:**

Controlled luxation was performed, followed by stiff rectangular mechanics, miniscrew-anchored mandibular distalization, and serial intraoral scan superimposition. After repeated bracket failure, a scan-guided customized lingual bracket pad was fabricated and bonded.

**Outcomes:**

After just over 5 months, the canine was derotated, uprighted, and positioned within the arch. Bilateral Class I occlusion, normal overjet and overbite, midline correction, and lower occlusal plane leveling were achieved. The incisor mandibular plane angle decreased from 122.5° to 104.4°. Electric pulp testing remained positive, and no additional mandibular incisor root resorption was detected compared with the referral baseline.

**Lessons:**

Serial intraoral scan superimposition may provide useful radiation-free evidence of post-luxation tooth movement, although registration error and crown-only assessment must be considered. A customized lingual bracket pad can improve bond reliability when heavy mechanics are required.

## 1. Introduction

Tooth ankylosis – the fusion of cementum or dentin to alveolar bone with obliteration of the periodontal ligament – occurs in both primary and permanent dentitions and carries meaningful functional and esthetic consequences, including infraocclusion, tipping of adjacent teeth, and alveolar remodeling.^[1]^ Reported prevalence varies widely because of differences in diagnostic criteria and populations; reviews cite ranges from 1% to 14% in primary teeth and lower rates in permanent teeth, and large imaging cohorts suggest that only a small fraction of impacted teeth show ankylosis with replacement resorption (2.6%). Age and tooth type also influence risk.^[2,3]^ If ankylosed teeth are not identified before orthodontic treatment, they will fail to respond to orthodontic forces and may instead cause displacement of adjacent teeth, occlusal plane canting, and secondary complications that are difficult to correct.^[4]^

Accurate diagnosis remains challenging because clinical signs, such as a high-pitched percussion note and absent mobility, are neither sensitive nor specific, and radiographic confirmation is imperfect.^[5]^ Although cone-beam computed tomography (CBCT) is often obtained to evaluate suspected ankylosis, it is not definitive; therefore, CBCT should be viewed as adjunctive rather than standalone.^[5]^ Moreover, CBCT reconstruction of crown surfaces is generally less faithful to fine external morphology than surface scanning, whereas intraoral scanners (IOSs) capture high-resolution surface geometry without ionizing radiation.^[6,7]^ Critically, radiographs are static snapshots; they cannot confirm whether a tooth is truly moving under orthodontic load. By contrast, serial IOS scans with best-fit superimposition enable radiation-free detection of translational and rotational changes over time.^[8]^ However, their accuracy depends on scanner precision, the registration algorithm, reference-area stability, and operator-defined masking.

Orthodontic management of ankylosed teeth has traditionally relied on several approaches, including surgical luxation with orthodontic traction, dentoalveolar distraction, segmental osteotomy, and, when repositioning proves unfeasible, decoronation or extraction with prosthetic replacement.^[1,9–11]^ These methods can sometimes achieve alignment or maintain alveolar volume, but they all share a critical limitation: clinicians must depend on indirect signs and static radiographs to determine whether true tooth movement has occurred. As a result, decisions such as whether repeat luxation is necessary, whether heavy mechanics are producing controlled movement, or whether distraction has been successful are often based on subjective or episodic assessments. In addition to conventional surgical-orthodontic options, adjunctive laser applications have been increasingly described because of their potential advantages in hemostasis, surgical precision, and postoperative comfort.^[12]^

Against this backdrop, this case report aimed to demonstrate a practical workflow that combines controlled luxation with serial IOS-based 3-dimensional (3D) superimposition to document orthodontic tooth movement in a partially ankylosed mandibular canine and to illustrate how IOS-enabled customization of a lingual bracket pad can improve bonding reliability under the heavy mechanics required in such cases.

## 2. Case presentation

### 2.1. Diagnosis and etiology

An adult female was referred by an orthodontist because leveling and alignment of the mandibular arch could not be achieved, with a working diagnosis of ankylosis involving the mandibular left canine. The previous orthodontist had attempted multiple strategies but was unable to upright or derotate the mandibular left canine. Her medical history was noncontributory.

**Figure 1.**
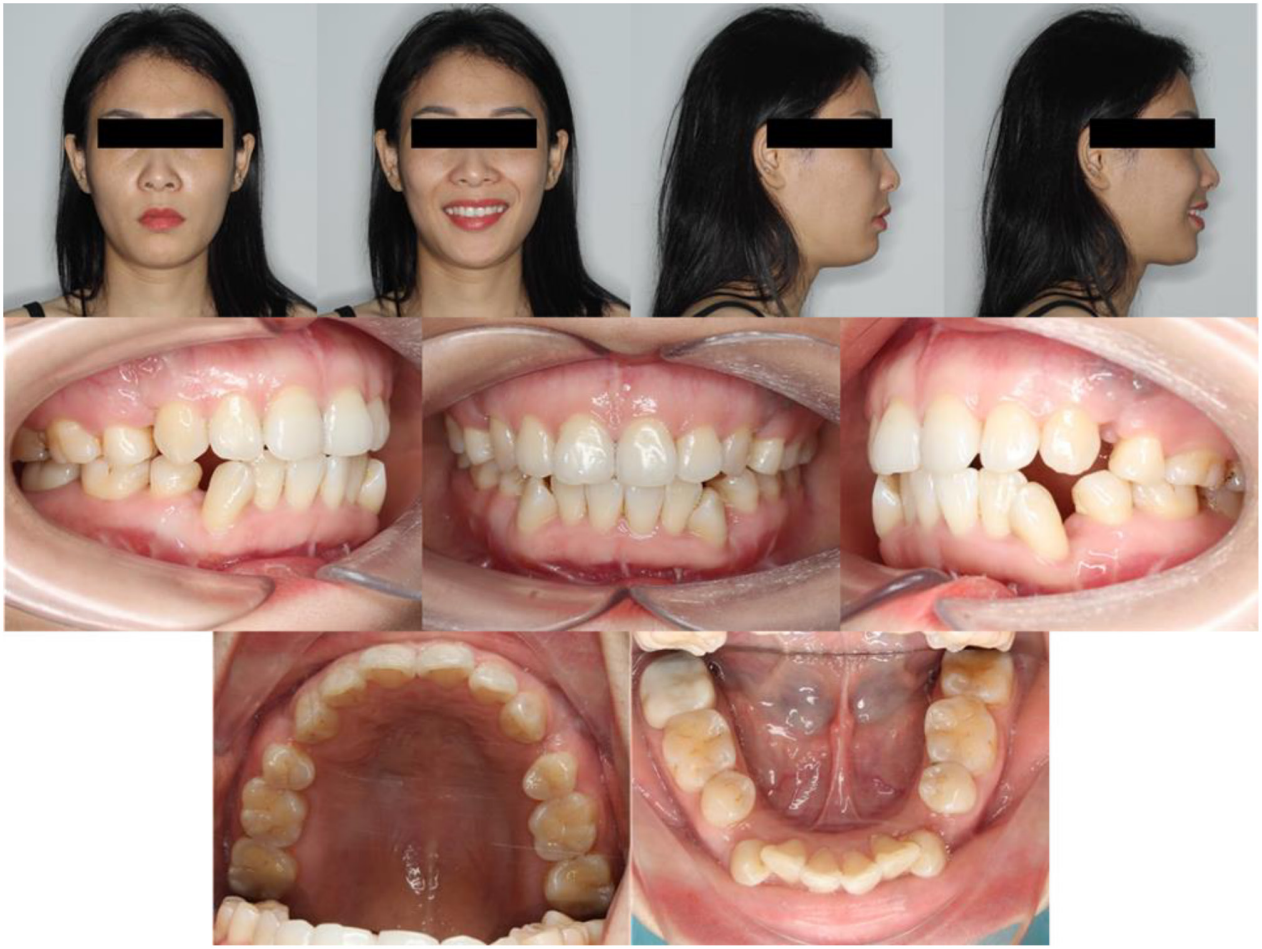
Pre-referral intraoral records.

**Figure 2.**
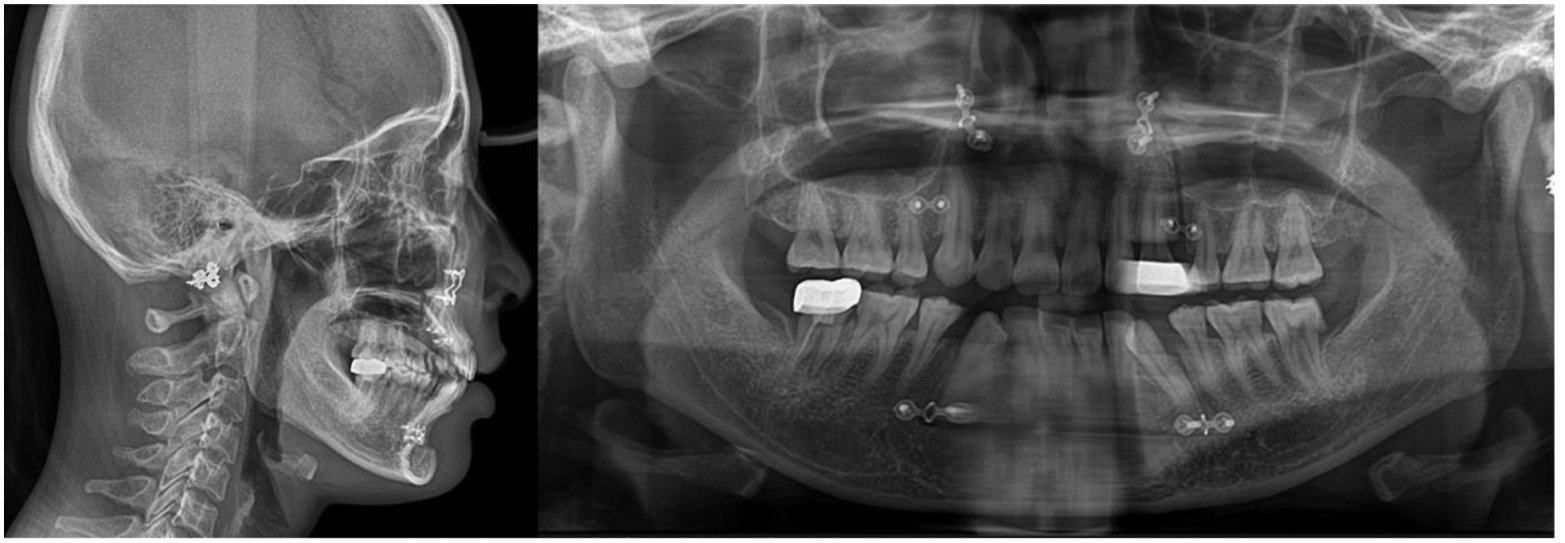
Pre-referral panoramic radiograph.

Dental history revealed that bimaxillary anterior segmental osteotomies with extraction of all first premolars had been performed 24 months before the current presentation to reduce dentoalveolar protrusion. Twenty-one months before referral, the patient began orthodontic treatment with the previous orthodontist to close the residual extraction spaces and complete occlusal detailing. At that time, clinical records documented approximately 3 mm spaces at each first-premolar extraction site, a slight Class III dental relationship, crowding in the mandibular arch with bilaterally severely rotated mandibular canines, and a 2-mm leftward deviation of the mandibular dental midline (Fig. 1). Panoramic radiographs additionally revealed incomplete consolidation along the osteotomy sites, with fixation screws and plates in situ. Both mandibular canines exhibited mesial inclination (Fig. 2). The patient was therefore bonded with a 0.018 × 0.025-inch lingual appliance (Linpass SL, 3B, Hangzhou, China), using a light-cure adhesive (White Glue, 3B, Hangzhou, China).

**Figure 3.**
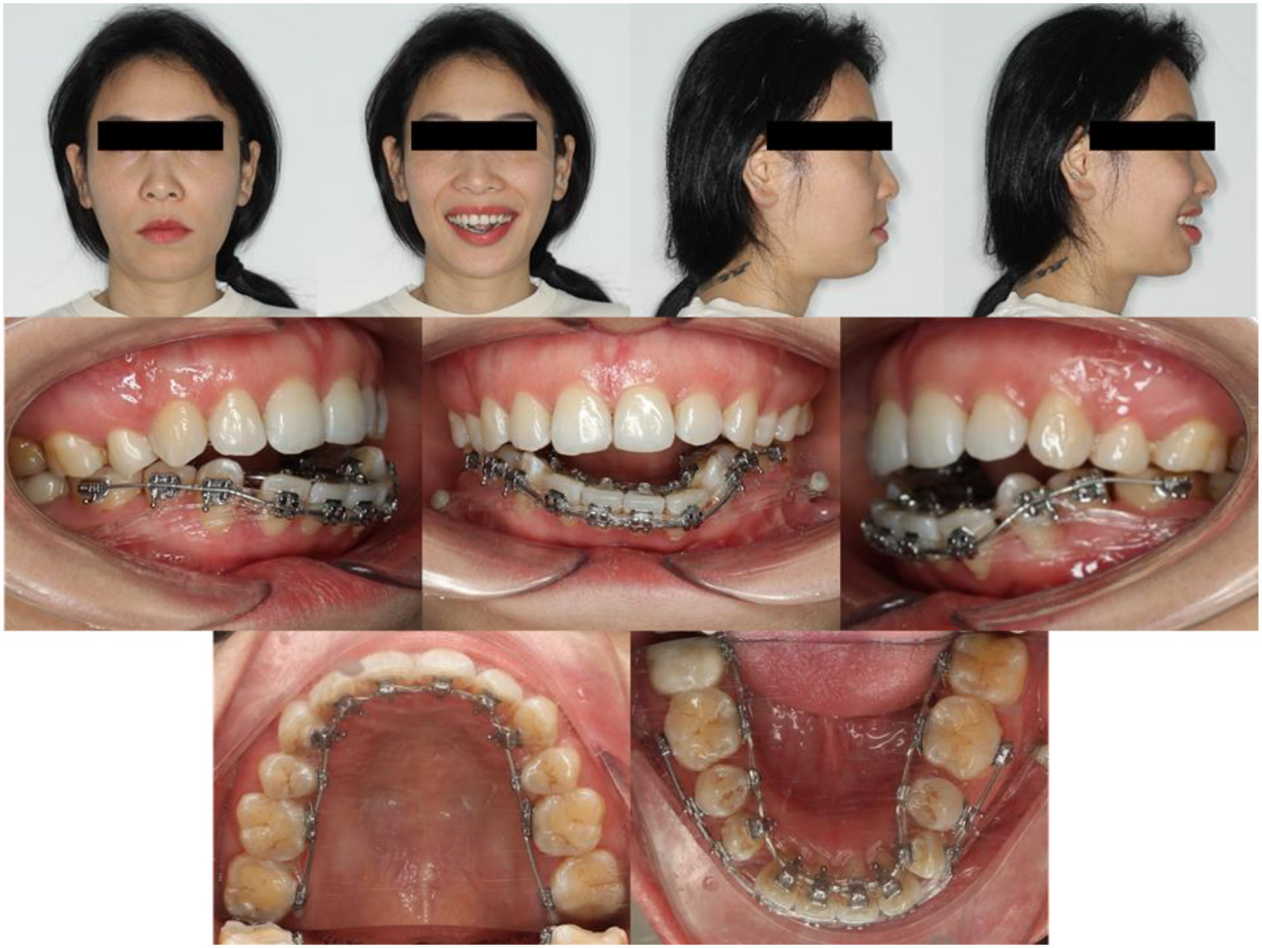
Extraoral photographs at presentation.

At the current examination, extraorally, the patient presented with a relatively harmonious facial appearance, a normal lower facial height, and a chin deviated slightly to the left, with an unesthetic smile due to a severe anterior open bite (Fig. 3). The profile was mildly convex. Intraorally, the patient was undergoing treatment with lingual brackets on both arches, with supplemental labial brackets (Trumpet, 3B, Hangzhou, China) bonded to the mandibular arch. Occlusal analysis revealed a slight Class III canine and molar relationship. A severe anterior open bite of 4.9 mm was evident, associated with a reverse curve of Spee in the mandibular arch and pronounced proclination of the lower incisors, resulting in a negative overjet of 3.8 mm. The mandibular left canine was severely rotated and failed to align despite prolonged orthodontic mechanics. Additionally, the mandibular right canine exhibited slight physiologic mobility, whereas the mandibular left canine showed none. The mandibular dental midline remained deviated more than 2 mm to the left, and residual extraction spaces were still present in the lower arch.

**Figure 4.**
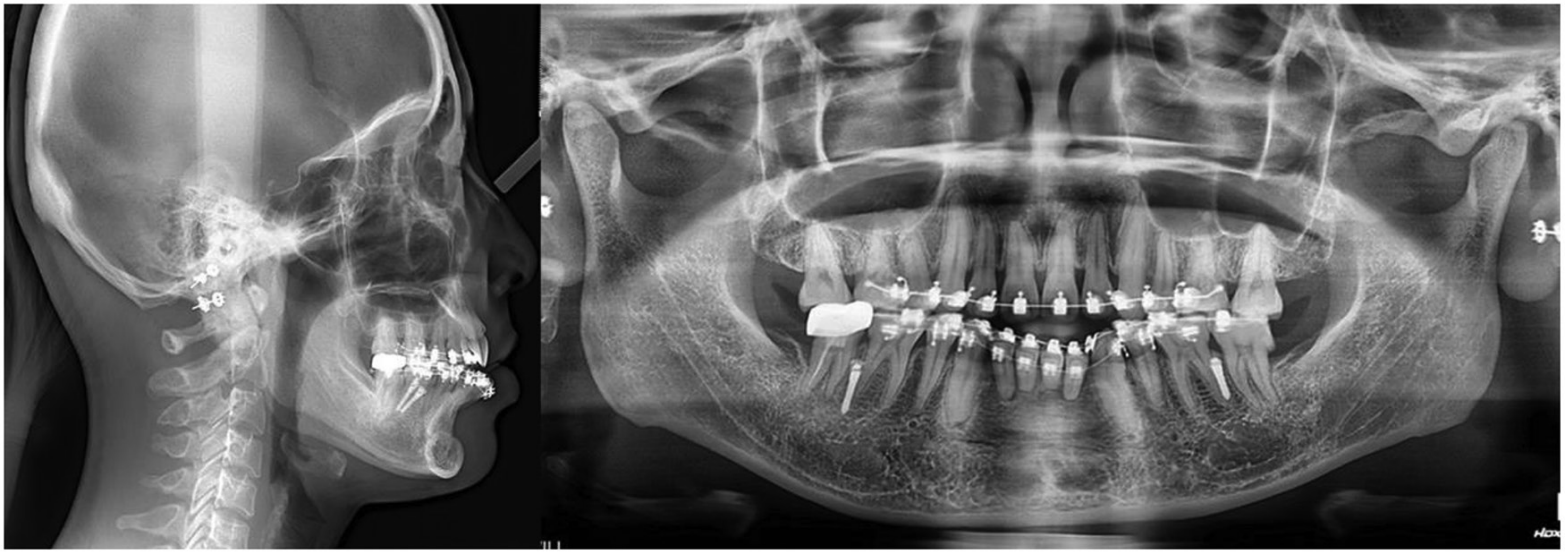
Panoramic radiograph at presentation.

Cephalometric analysis demonstrated a skeletal Class II pattern with an A-point–nasion–B-point angle of 5.0°, attributable to a retrognathic mandible with a sella–nasion–B-point angle of 73.8°, and a normodivergent vertical pattern with a Frankfort–mandibular plane angle (FMA) of 27.8° (Table 1). The maxillary incisors were slightly retroclined with an upper incisor–sella–nasion (U1–SN) angle of 92.9°, whereas the mandibular incisors were severely proclined with an incisor mandibular plane angle of 122.5°. The lower lip was 2.2 mm anterior to the esthetic line, confirming protrusion beyond the normal range due to the flared mandibular incisors. A panoramic radiograph confirmed the removal of all fixation plates; however, the screw-removal bony defect in the left mandible projected over the apex of the mandibular left canine, with a segmentally discontinuous periodontal ligament space. Both mandibular canines showed improved mesial inclination (Fig. 4). In addition, the mandibular incisors exhibited short roots, which may be attributed to their pronounced labial proclination (causing a foreshortened radiographic projection) and root resorption induced by reactive forces generated during attempts to align the mandibular left canine.

**Table 1:** Cephalometric measurements.

Measurements	Norm, mean ± SD	At presentation	Posttreatment	Change
Skeletal				
SNA (°)	81.2 ± 3.7	78.8	78.9	0.1
SNB (°)	79.2 ± 3.8	73.8	72.9	−0.9
ANB (°)	2.5 ± 1.8	5	6	1
FMA (°)	25.0 ± 4.0	27.8	27.3	−0.5
Dental				
U1-SN (°)	105.3 ± 6.6	92.9	94.2	1.3
IMPA (°)	90.0 ± 3.5	122.5	104.4	−18.1
U1-NA (°)	22.0 ± 5.0	14.1	15.3	1.2
U1-NA (mm)	4.0 ± 3.0	2.8	2.1	−0.7
L1-NB (°)	25.0 ± 5.0	53.2	34.5	−18.7
L1-NB (mm)	4.0 ± 2.0	14	10	−4.0
Upper incisal display (mm)	2.5 ± 1.5	1.7	3.1	1.4
Overjet (mm)	2.0 ± 2.0	−3.8	1.3	5.1
Overbite (mm)	2.0 ± 2.0	−4.9	0.9	5.8
Soft tissue				
E-line–upper lip (mm)	0.0 ± 2.0	−0.6	−0.1	0.5
E-line–lower lip (mm)	0.0 ± 2.0	2.2	1.7	−0.5
Extraction index	153.8 ± 7.8	150.3	153.5	3.2

ANB = A-point–nasion–B-point angle, E-line = esthetic line, FMA = Frankfort–mandibular plane angle, IMPA = incisor mandibular plane angle, L1 = mandibular central incisor, NA = nasion–A-point line, NB = nasion–B-point line, SD = standard deviation, SN = sella–nasion plane, SNA = sella–nasion–A-point angle, SNB = sella–nasion–B-point angle, U1 = maxillary central incisor.

The patient was diagnosed with mild Class III malocclusion on a skeletal Class II base with a severe anterior open bite, negative overjet, and partial ankylosis of the mandibular left canine, likely associated with prior osteotomy and fixation screw impingement.

### 2.2. Treatment objectives

The treatment objectives were to confirm whether the mandibular left canine could be mobilized after controlled luxation; derotate, upright, and align the canine within the mandibular arch; close the residual extraction spaces and correct the mandibular dental midline; resolve the anterior open bite and negative overjet; and improve mandibular incisor inclination while minimizing additional root resorption.

### 2.3. Treatment alternatives

The possible treatment options were carefully considered. The first option was extraction of the mandibular left canine with orthodontic space closure, which would remove the suspected ankylosed tooth and simplify the mechanics for leveling and alignment. However, eliminating a mandibular canine would markedly increase the mandibular tooth-size deficiency relative to the maxillary dentition, making it difficult to achieve normal overjet and coordinated interdigitation without additional compensations, such as substantial maxillary reduction or restorative augmentation.

The second option was to bypass the mandibular left canine, align and level the remaining teeth, and plan prosthetic replacement of the canine after orthodontic treatment. However, this strategy would leave the left-sided Class III dental relationship uncorrected during treatment, and the “parked” canine could mechanically interfere with the required retraction of the mandibular incisors to resolve the crossbite and normalize the overjet.

The third option was to continue with carefully controlled orthodontic mechanics, aided by surgical luxation of the mandibular left canine to overcome the suspected ankylosis. The principal risk of this method was pulpal necrosis with possible downstream complications. After counseling on the risks and benefits, the patient chose the third option. However, if tooth movement could not be objectively confirmed, treatment could be unnecessarily prolonged, leading to sustained reactive forces that increase the risk of further root resorption in adjacent teeth. To avoid this, a strict monitoring protocol was established using serial intraoral scans with 3D superimposition. This novel approach enables the detection of subtle, millimeter-scale displacements of the mandibular left canine relative to regional control teeth, thereby providing early confirmation of effective tooth movement.

### 2.4. Treatment progress

**Figure 5.**
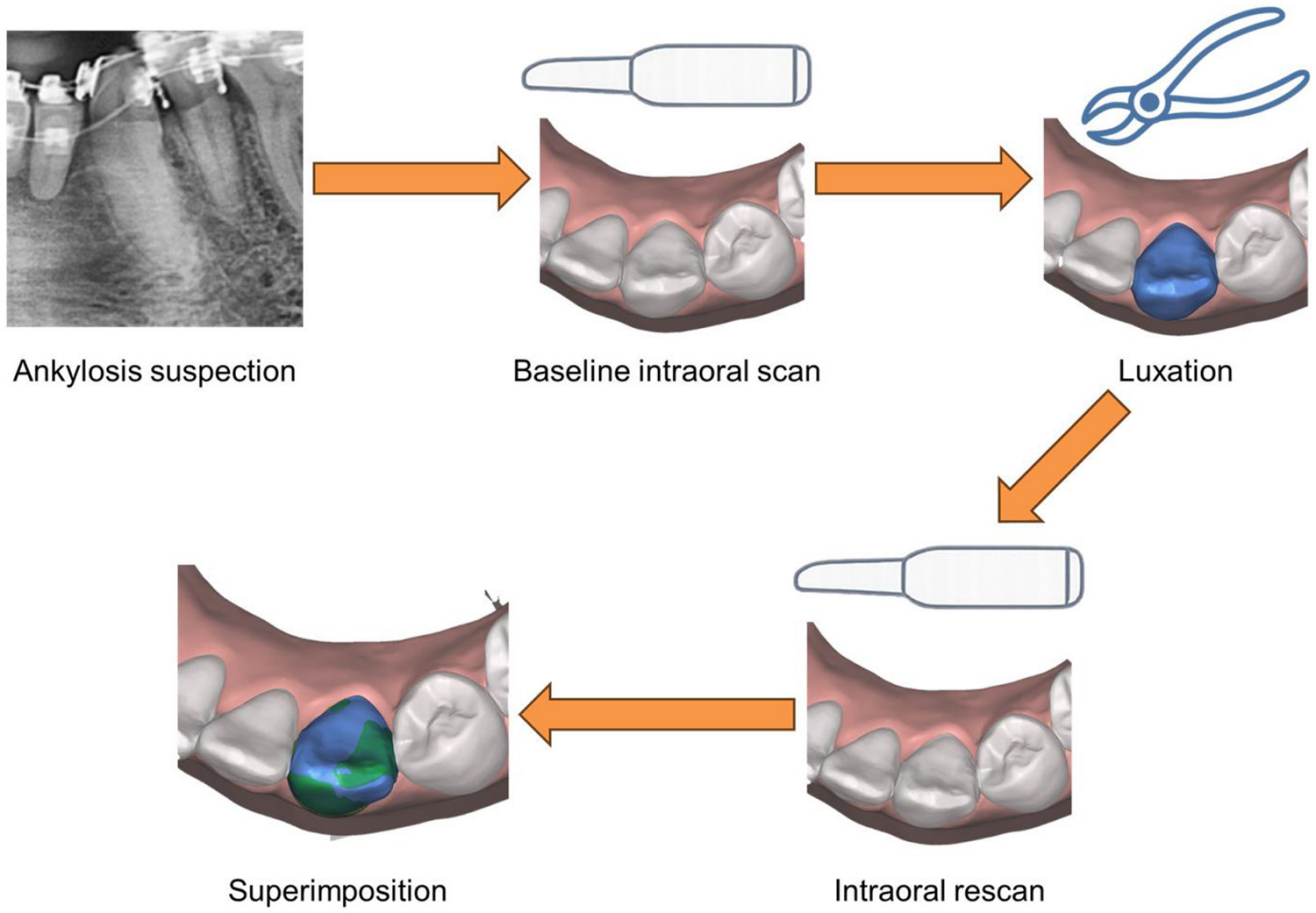
Workflow of superimposition for monitoring an ankylosed mandibular canine. A baseline intraoral scan was obtained, followed by luxation of the ankylosed canine, a repeat intraoral scan, and subsequent best-fit superimposition to document post-luxation displacement.

At baseline, the lower arch was engaged with 0.016 × 0.022-inch lingual and 0.017 × 0.025-inch labial nickel–titanium (NiTi) archwires. A reference full-arch intraoral scan was acquired with an i600 scanner (Medit, Seoul, Korea) for subsequent superimposition. This initiated our mobility-screening protocol for the suspected ankylosed canine, in which follow-up scans were rigidly registered to the baseline scan, and millimeter-scale displacement beyond repeatability thresholds was quantified to confirm true movement before escalating mechanics or repeating luxation (Fig. 5). Under local anesthesia, surgical luxation of the mandibular left canine was performed to disrupt the suspected ankylotic interface while simultaneously improving its derotation. The tooth was grasped at the cervical portion of the crown, avoiding any contact with the brackets, to minimize the risk of bracket debonding. The lower lingual arch was then advanced to 0.017 × 0.025-inch NiTi, and the labial arch to 0.019 × 0.025-inch NiTi, to help maintain the post-luxation position and deliver rectangular control. Concurrently, miniscrew-anchored forces were applied to distalize the mandibular dentition to address the Class III dental relationship.

**Figure 6.**
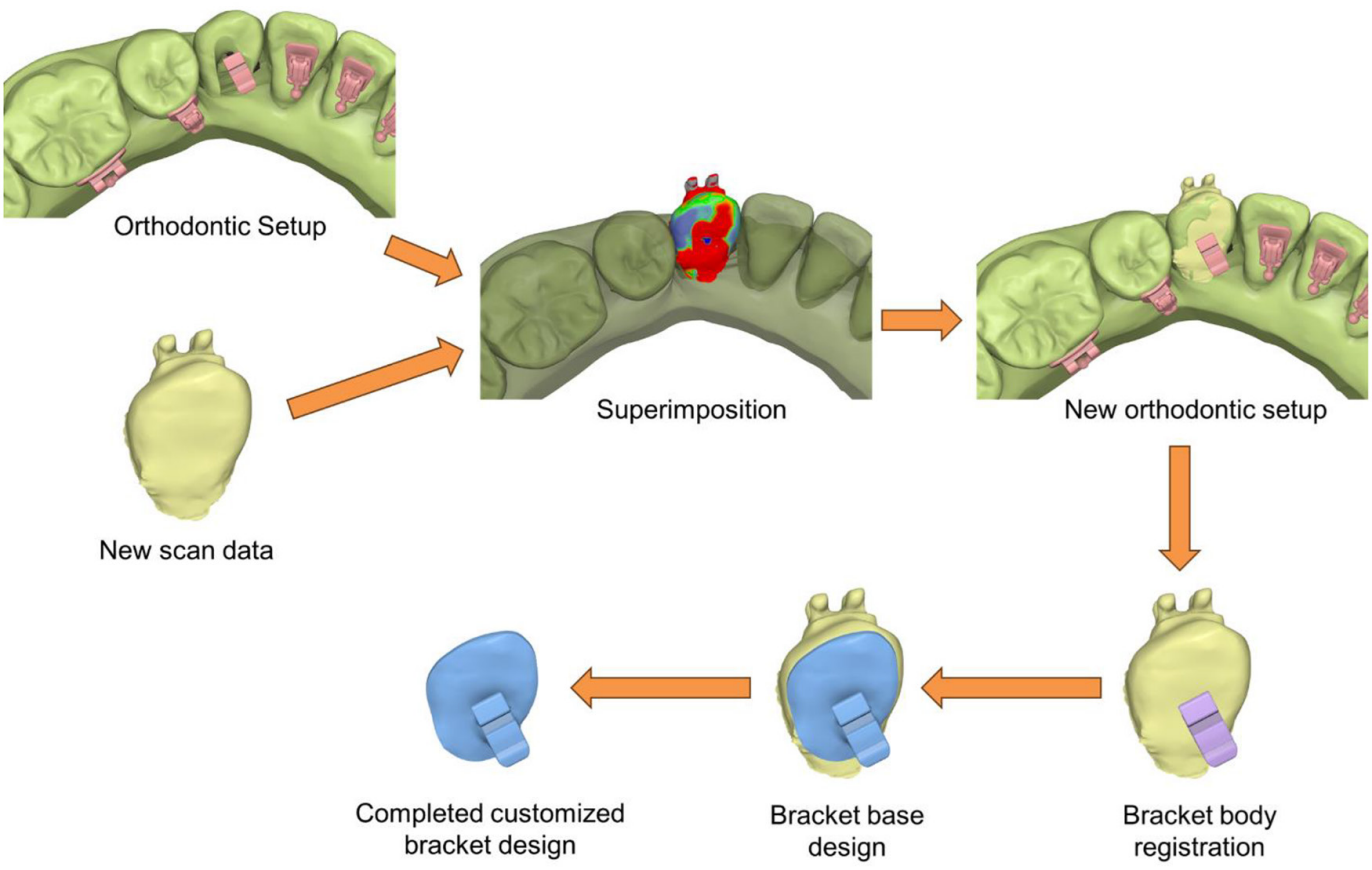
Intraoral-scanning workflow for customized lingual bracket fabrication: segmentation of the new canine scan; constrained alignment to the incisal ridge and labial surface (lingual excluded); pad design; combination of the pad with the registered bracket body to create a complete customized bracket; 3D-printed wax and cast.

However, the increased wire stiffness and force levels precipitated repeated debonding of the stock lingual bracket on the mandibular left canine, with multiple rebonding attempts required during the subsequent month. To restore reliable control, the newly exposed enamel surface of the mandibular left canine was rescanned with the same IOS. The canine crown was segmented from this new scan and superimposed onto the patient’s existing mandibular orthodontic setup by constraining the best-fit alignment to the incisal ridge and labial surface only, deliberately excluding the lingual surface to avoid bias from the previous pad morphology. Because the canine had been severely rotated at baseline, the pretreatment scan did not permit accurate customization of the lingual pad. Using the updated, constrained registration, a customized lingual bracket pad was designed using Meshmixer software (Autodesk, San Rafael) (Fig. 6). A wax pattern was 3D printed using a Saturn S printer (Elegoo, Shenzhen, China) and cast in a nickel-chromium alloy (VeraBond, Aalbadent, Fairfield). The customized bracket was bonded, and a 0.016 × 0.022-inch lingual NiTi archwire was reengaged.^[13]^

At each visit, serial lower-arch scans were obtained and rigidly registered to the reference scan using best-fit surface registration (iterative closest-point) constrained to a mandibular posterior mask comprising the bilateral first and second molars and second premolars. The mandibular left canine and the adjacent extraction site were excluded from the registration region. Displacement of the mandibular left canine was quantified relative to this posterior reference segment, providing early, objective confirmation of effective movement and guiding timely adjustments. Serial superimposition and masking were performed using Medit Design software (Medit, Seoul, Korea).

**Figure 7.**
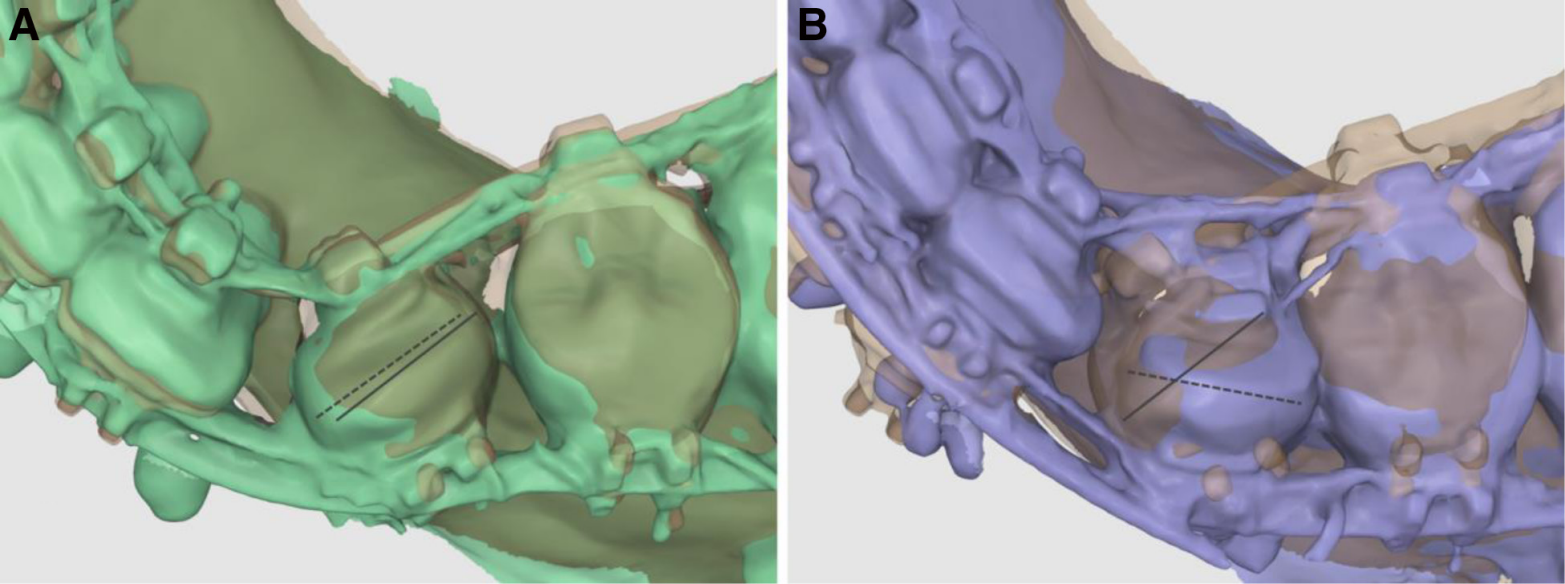
Serial intraoral scan superimposition. (A) One month (green) versus baseline (beige); (B) 2.5 months (purple) versus baseline (beige).

**Figure 8.**
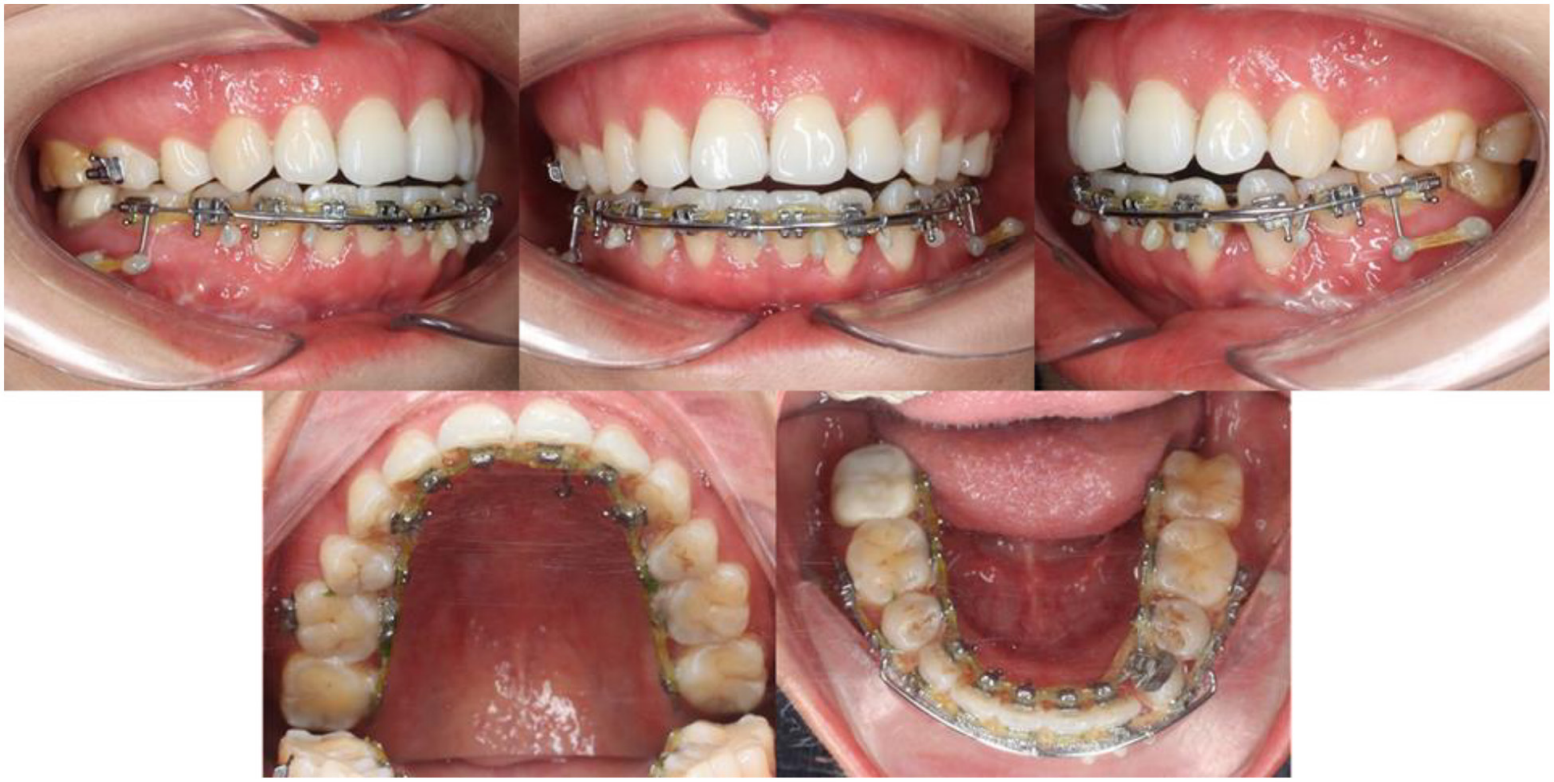
Follow-up superimposition after second luxation.

One month after rebonding, superimposition demonstrated a slight 1.9° derotation of the mandibular left canine, indicating early responsiveness (Fig. 7). The lingual wire was advanced to 0.017 × 0.025-inch NiTi, and a second luxation was performed. Additionally, a heavy auxiliary expansion arch was inserted into the lower arch to improve arch coordination because the partial ankylosis had contributed to a reduced mandibular intercanine width compared with the maxillary intercanine width. At the subsequent 6-week review, serial superimposition showed a marked 45.1° derotation of the partially ankylosed canine, and physiologic mobility was also observed; therefore, no further luxation was undertaken (Fig. 8). Treatment then proceeded with continued space closure and mandibular distalization. One month later, the lower lingual arch was progressed to a rigid 0.017 × 0.025-inch stainless-steel wire, and the labial arch was progressed to a 0.019 × 0.025-inch stainless-steel wire, and intermaxillary elastics were introduced for finishing and occlusal detailing. After an additional 2 months, the appliances were removed, and fixed retainers were bonded in both arches.

### 2.5. Treatment results

**Figure 9.**
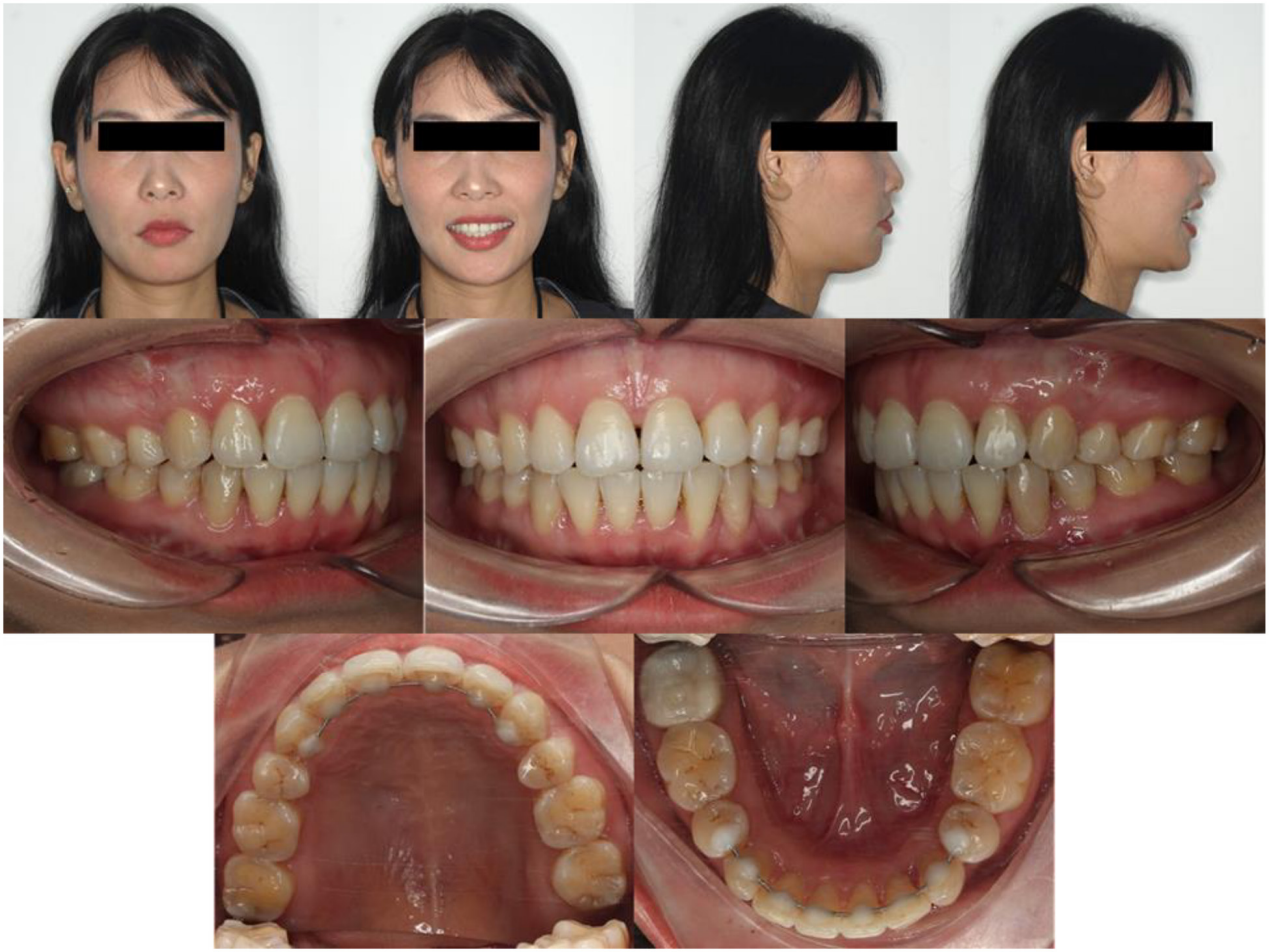
Posttreatment extraoral photographs.

After just over 5 months of treatment using a novel protocol of controlled luxation with strict IOS-based monitoring and 3D superimposition, the patient showed marked improvements. Extraorally, the patient exhibited a noticeably improved smile esthetics as the anterior open bite was successfully closed. Retraction of the mandibular incisors produced a softer lower lip profile, resulting in a more balanced facial appearance (Fig. 9).

Intraorally, a stable bilateral Class I canine and molar relationship was achieved. The mandibular midline was corrected to coincide with the maxillary midline, and normal overjet and overbite were established. The mandibular left canine was successfully derotated, uprighted, and expanded, contributing to proper arch coordination. The lower occlusal plane was leveled, and all extraction spaces were completely closed.

**Figure 10.**
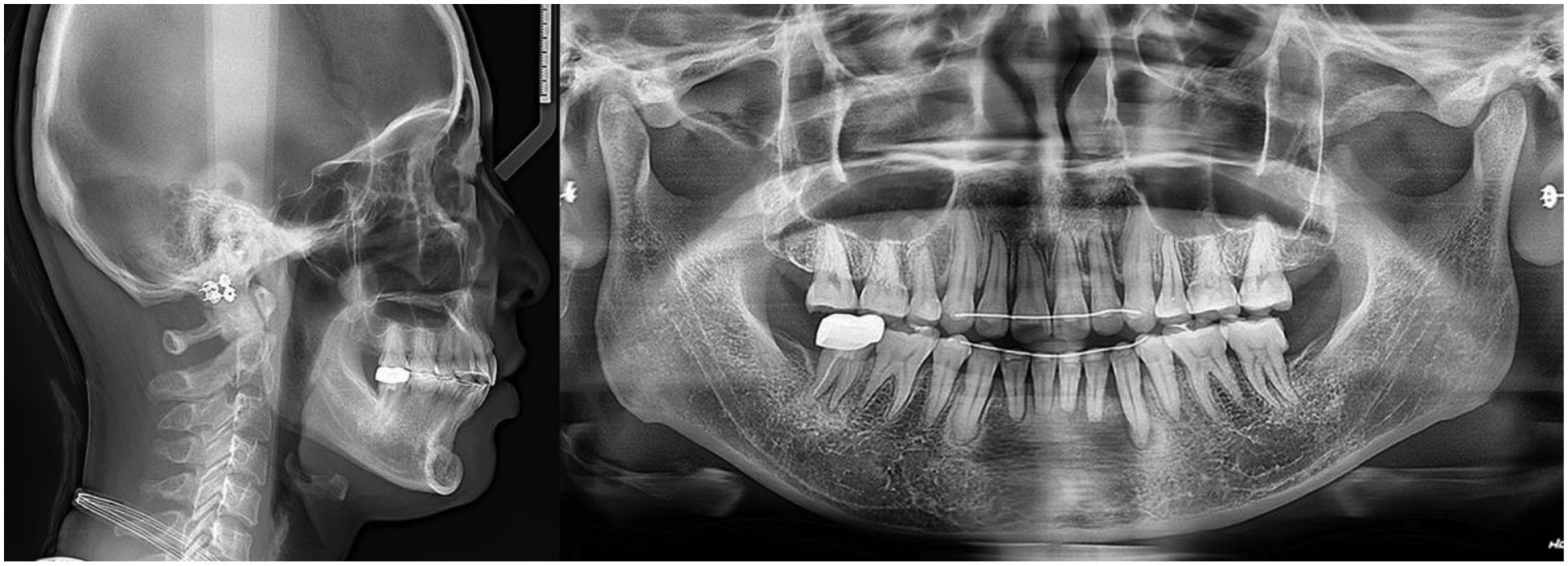
Posttreatment cephalogram and tracings.

Posttreatment cephalometric evaluation demonstrated that the overall skeletal relationship was largely maintained. The inclination of the maxillary incisors showed slight improvement (U1–SN, 94.2°), while the mandibular incisors were significantly retracted (incisor mandibular plane angle, 104.4°). These dental movements led to a favorable improvement in soft tissue balance, with the lower lip positioned 1.7 mm anterior to the esthetic line, indicating a more harmonious profile (Fig. 10). Panoramic radiography showed no qualitative evidence of additional mandibular incisor root resorption compared with the referral baseline. The apparent increase in root length was attributed to reduced foreshortening after incisor uprighting rather than true root elongation. The mandibular left canine exhibited a transient widening of the periodontal ligament space consistent with the large magnitude of movement over a short interval; however, the tooth remained positive on electric pulp vitality testing and was managed with close follow-up.

## 3. Discussion

This case report presents the management of a partially ankylosed mandibular left canine using controlled luxation together with serial intraoral scan-based 3D superimposition to document orthodontic tooth movement and an IOS-enabled customized lingual bracket pad designed and fabricated to ensure reliable delivery of heavier mechanics when needed. Because ankylosis is difficult to diagnose and monitor with certainty, we employed best-fit surface registration on a mandibular posterior reference mask to detect millimeter-scale translational change and derotation of the target tooth in a chairside workflow. Early objective confirmation of movement allowed timely adjustment of forces, shortening ineffective intervals and reducing collateral risks from prolonged reactive forces on adjacent teeth. The scan-driven customization – segmenting the newly scanned canine crown and constraining alignment to the incisal ridge and labial surface while deliberately excluding the lingual surface – improved bond reliability after repeated debonding under heavier rectangular wires. Clinically, the combined strategy enabled the partially ankylosed mandibular left canine to be derotated, uprighted, and translated into its planned position within the arch in just over 5 months, with preserved pulpal vitality and no additional incisor root resorption. As a result, orthodontic finishing, arch coordination, and the establishment of bilateral Class I relationships with normalized overjet and overbite were achieved. To our knowledge, this is the first in vivo study combining controlled luxation with serial intraoral scan-based 3D superimposition to document post-luxation mobility, together with an intraoral scan-enabled customized lingual bracket pad to ensure reliable bonding under heavy mechanics.

A potential advantage of IOSs in monitoring suspected tooth ankylosis is their nonionizing, high-resolution, 3D surface capture, which may reduce repeated radiographic exposure during short-interval follow-up.^[14,15]^ Nevertheless, IOS-based monitoring should be considered an adjunct, not a replacement, for clinical examination and radiographic assessment. CBCT remains important for evaluating root position, alveolar bone, periodontal ligament space, and replacement resorption, although it may produce false-positive or equivocal findings in ankylosis diagnosis.^[3,6,16]^ In contrast, IOS records only crown-surface morphology and cannot directly visualize the periodontal ligament, root surface, alveolar bone, or ankylotic interface. Therefore, the present workflow should not be interpreted as a direct diagnostic method for ankylosis but as a surface-based approach for documenting positional change after luxation and orthodontic loading.

The interpretation of serial IOS superimposition is also vulnerable to registration error. Accuracy depends on scanner precision, scan-path consistency, the software algorithm, reference-area stability, operator-defined masking, saliva or soft-tissue artifacts, and biologic changes in the reference teeth. Although the mandibular left canine and adjacent extraction site were excluded from the registration mask to reduce bias, small displacement values should still be interpreted cautiously and correlated with clinical mobility, occlusal changes, and radiographic findings. Further prospective studies with larger cohorts, standardized registration protocols, repeated-scan error analysis, and long-term follow-up are needed before this workflow can be recommended for broader clinical adoption.

Surgical luxation followed by orthodontic traction is well documented for realigning ankylosed teeth, with short-term alignment often achievable. However, reported sequelae include pulp canal obliteration, calcific metamorphosis, pulpal necrosis, external replacement resorption, root resorption, and re-ankylosis.^[10,17,18]^ Our case belongs to this therapeutic familyyet adds a methodological advance that is generally absent from previous luxation reports by providing objective, radiation-free verification of post-luxation mobility via serial intraoral scan best-fit superimposition. This novel approach allowed millimeter-scale confirmation of derotation and translation under known mechanics and informed timely adjustments rather than relying solely on periodic clinical or radiographic impressions. By quantifying the magnitude of displacement, it provided an objective basis to support clinical decision-making on whether repeat luxation was warranted or could be avoided.

By comparison, dentoalveolar distraction and segmental (single-tooth) osteotomy move a tooth–bone block and can reposition severely infrapositioned teeth, but they are more invasive, device-dependent, and costlier.^[1,11]^ Furthermore, these reports typically depend on intermittent imaging rather than continuous, fine-scale tracking. When repositioning is impractical, particularly in growing patients, decoronation preserves ridge volume for future prosthetics, and in adults, extraction with prosthetic replacement or orthodontic space closure remains reasonable on a case-by-case basis.^[19,20]^ However, prosthetic replacement is generally less desirable than preserving the natural tooth when feasible, as it entails additional cost and long-term maintenance and, in growing patients, carries a well-documented risk of infraocclusion.^[21]^ Overall, the evidence base consists largely of case reports and small series, with no randomized trials comparing interventions, so treatment selection hinges on biologic plausibility and clinician judgment.^[22]^

A practical commonality across luxation-assisted alignment and dentoalveolar distraction is the use of stiff rectangular mechanics to stabilize the tooth immediately after mobilization and to deliver decisive movement before re-ankylosis can occur. In addition, robust rectangular mechanics elicit a pronounced aseptic inflammatory response in the periodontal ligament – with upregulation of cytokines (e.g., interleukin-1β and tumor necrosis factor-α) and osteoclast-mediated remodeling – that, together with early activation, is thought to help limit re-ankylotic bridging, although higher force levels also increase the risk of root resorption.^[9,23–25]^ Following luxation, early activation is recommended to prevent re-ankylosis; several reports describe immediate traction and stabilization with rigid archwires after mobilization or repositioning, emphasizing the need for stable mechanics while periodontal healing proceeds.^[4,26]^ Dentoalveolar distraction and single-tooth osteotomy similarly rely on robust force systems (device-driven or wire-mediated) to translate a tooth–bone block rapidly, again prioritizing stability during the early postsurgical interval.^[27]^ Accordingly, heavy rectangular mechanics are integral to both post-luxation protocols and distraction and segmental osteotomy workflows to maintain the newly achieved position, deliver early controlled movement, and minimize the window for re-ankylosis or relapse.

In this context, heavy mechanics bring a practical vulnerability: bracket debonding can negate the positional gain and permit relapse toward the pre-luxation position. Clinical series show nontrivial debond rates – particularly in the lower arch and during early treatment – underscoring the need for reliable bonding when high forces and large rectangular wires are used. Customized brackets and pads can improve bond reliability by optimizing the bonding interface rather than merely increasing retention. In the present case, IOS-derived segmentation allowed the design of an enlarged, close-fitting lingual pad, thereby increasing the enamel–bracket contact area, improving base adaptation, and reducing excessive adhesive thickness. This is clinically relevant because an overly thick resin layer may become the mechanically weakest part of the enamel–adhesive–bracket complex.^[28,29]^ In vitro studies have shown that customized orthodontic brackets, including lingual designs with enlarged bases, can achieve debonding forces comparable to or greater than those of conventional brackets when base fit and bonding area are optimized.^[30,31]^ Equally important, meticulous isolation was required during rebonding because blood, saliva, and certain pretreating agents have been reported to reduce orthodontic bracket bond strength.^[32–34]^ Thus, the scan-guided customized pad, combined with careful contamination control, helped restore reliable force delivery after repeated debonding and permitted the use of heavier rectangular mechanics required for movement of the previously ankylosed canine. Contemporary evidence also supports the role of IOS-based virtual setups in improving the accuracy of customized appliance fabrication and bracket placement.^[35–37]^

In this case, the lower incisor proclination and anterior open bite were interpreted as primarily iatrogenic, resulting from prolonged reactive mechanics against the mesially tipped ankylosed canine rather than tongue-thrust–related dysfunction; therefore, no formal logopedic or myofunctional therapy was indicated.

This study has several limitations. This is a single-case report without a control; therefore, causal inferences and generalizability are limited, and the relatively short active treatment and follow-up period cannot establish long-term stability or exclude late sequelae such as pulpal necrosis, re-ankylosis, periodontal remodeling, occlusal relapse, or loss of mobility. The IOS workflow documents crown-surface displacement only; it cannot visualize the periodontal ligament or roots, so crown movement may not fully reflect apical/torque changes. Surface-based best-fit superimposition assumes a stable posterior reference segment and is susceptible to registration error, scanner precision limits, saliva and soft-tissue artifacts, and operator-dependent masking choices. The customized lingual pad procedure depends on specific hardware, software, and laboratory support, which may limit external reproducibility. Finally, concurrent mechanics, including miniscrew-anchored distalization, expansion, and the use of heavy rectangular wires, are potential confounders of the rate and pattern of movement. Prospective studies with larger cohorts, predefined registration protocols, repeated-scan error analysis, and long-term clinical and radiographic outcomes are needed before this workflow can be recommended for broad clinical adoption.

## 4. Conclusion

Combining controlled luxation with serial IOS-based 3D superimposition allowed radiation-free, millimeter-scale confirmation of post-luxation mobility and timely modulation of forces. An IOS-enabled customized lingual pad improved bonding reliability under heavy mechanics, preserving positional gains. Clinically, the partially ankylosed mandibular left canine was derotated, uprighted, and positioned within the arch in just over 5 months, with preserved pulpal vitality and no additional mandibular incisor root loss versus the referral baseline. Larger prospective studies are warranted to assess reproducibility, quantify superimposition error, and compare this workflow with alternatives such as dentoalveolar distraction or single-tooth osteotomy.

## Author contributions

**Conceptualization:** Viet Anh Nguyen.

**Data curation:** Viet Anh Nguyen.

**Formal analysis:** Viet Anh Nguyen.

**Funding acquisition:** Viet Anh Nguyen.

**Investigation:** Viet Anh Nguyen, Thi Hanh Pham.

**Methodology:** Viet Anh Nguyen, Thi Hanh Pham.

**Project administration:** Viet Anh Nguyen, Anand Marya.

**Resources:** Viet Anh Nguyen, Viet Hoang.

**Software:** Viet Anh Nguyen, Viet Hoang.

**Supervision:** Viet Anh Nguyen.

**Validation:** Viet Anh Nguyen.

**Visualization:** Viet Anh Nguyen.

**Writing – original draft:** Viet Anh Nguyen, Anand Marya.

**Writing – review & editing:** Viet Anh Nguyen, Tim Joda, Hang Nga Mai, Viet Hoang, Anand Marya.
